# Multiphysics Investigation on Thermal Characteristics of Internal Bio-Inspired V-Ribbed Cooling Channels for Outer Rotor PMSM

**DOI:** 10.3390/biomimetics11060441

**Published:** 2026-06-22

**Authors:** Xin Xiong, Xiangyu Li, Shawn You, Bing Zhu, Ping Ding, Huanhuan Gao, Zongqi Hou

**Affiliations:** 1 School of Automotive Engineering, Yancheng Institute of Technology, Yancheng 224000, China; xiongxin@ycit.edu.cn (X.X.); zhubing@ycit.edu.cn (B.Z.); dingping@stu.ycit.edu.cn (P.D.); gaohuanhuan@stu.ycit.edu.cn (H.G.); houzongqi@stu.ycit.edu.cn (Z.H.); 2 Chongqing Institute of High-Tech Industry, Chongqing 404100, China; shawnyou@globalevbpc.com

**Keywords:** outer rotor PMSM, bio-inspired cooling, multiphysics analysis, thermal management

## Abstract

Meeting the rigorous performance standards of modern electrified transit necessitates the deployment of high-performance outer rotor PMSMs with elevated power-to-volume ratios. However, their unique internal heat source topology inherently restricts heat dissipation. This limitation risks permanent magnet demagnetization and winding insulation failure. To address these thermal bottlenecks, this paper proposes internal bio-inspired cooling channels. These channels feature micro-scale V-shaped ribs. This design targets a 60 kW outer rotor PMSM. The motor uses a fractional-slot concentrated winding. The analytical procedure commences with the formulation of a transient 2D numerical model utilizing the Time-Stepping Finite Element approach (TS-FEM). It is coupled with the Bertotti model to compute electromagnetic losses. This approach accurately determines losses under high-frequency rated conditions. Results reveal that stator iron loss constitutes the dominant heat source. It accounts for 76.4 percent of the total electromagnetic loss. Furthermore, these losses show severe spatial concentration at the stator teeth. Subsequently, a three-dimensional fluid-solid coupled CFD model is developed. This model evaluates the proposed internal cooling channels. The design integrates bio-inspired vein networks and V-shaped ribs. These internal ribs disrupt the near-wall thermal boundary layer. This disruption enhances the local convective heat transfer. Comparative multiphysics analyses indicate improved hydraulic and thermal performance of the bio-inspired design under the same numerical boundary conditions. The bio-inspired channel achieves a more uniform static pressure distribution and reduces severe fluid stagnation zones. In the numerical model, the maximum stator and permanent magnet temperatures are reduced to 48 °C and 42 °C, respectively. This work provides a numerical design reference for thermal management in high-performance electric aviation.

## 1. Introduction

In the context of global carbon neutrality, the electrification of transportation and industrial drive systems has been widely promoted. Characterized by enhanced torque-to-volume ratios and a space-efficient axial profile, the outer-rotor PMSM facilitates direct-drive operations, leading to its extensive adoption in both the automotive industry and the rapidly evolving field of electric aerospace. However, the outer rotor topology presents unique thermal challenges compared to inner rotor motors. The heat-generating stator is enclosed inside the rotor. This structural configuration makes heat dissipation inherently difficult. Moreover, the direct-drive operation often involves complex duty cycles. Significant heat accumulation is triggered by the combination of restricted internal volumes and high-power densities. Such elevated temperatures jeopardize the integrity of the permanent magnets (PMs) by risking irreversible thermal demagnetization. They also threaten the insulation integrity of the windings. These combined thermal issues compromise energy efficiency and system safety. Consequently, achieving high-fidelity thermal field estimation is of paramount importance. Furthermore, the refinement of an innovative hybrid cooling architecture remains indispensable for the long-term viability of high-efficiency outer-rotor PMSM designs.

In recent studies, the critical nature of electromagnetic losses and their impact on thermal profiling have been widely examined [1,2,3,4,5,6,7,8,9,10,11,12]. While various analytical and numerical frameworks—including equivalent thermal networks [13,14] and finite element simulations [15]—have successfully mapped the thermal characteristics of motor components, these studies frequently assumed an ideal balance between heat generation and dissipation, leaving the transient temperature field underexplored. This specific field must account for the highly complex loss distribution inherent to outer rotor motors. Consequently, the current investigation leverages established theoretical foundations to conduct a detailed assessment of both transient and steady-state thermal profiles, specifically accounting for the influence of electromagnetic harmonics [16,17].

A reasonable cooling structure design is the main factor affecting PM motor heat dissipation. Therefore, this topic has rapidly attracted widespread scholarly attention. Nevertheless, standard cooling configurations frequently prove inadequate for the thermal requirements of high-power-density outer-rotor machines. These methods include serial spiral channels and axial Z-type channels. Spiral channels tend to result in significant axial temperature gradients. This issue occurs due to coolant saturation along the lengthy flow path. Meanwhile, Z-type channels often incur excessive hydraulic pressure penalties. Flow separation at sharp bends causes these specific penalties. To resolve these contradictions, biomimetics has provided novel inspiration for thermal engineering. Through millions of years of evolution, nature has developed optimal structures for fluid transport and thermal management. Specifically, insect wing vein networks exhibit enhanced capabilities in macro-scale fluid distribution [18]. These structures achieve minimal pressure drop and effectively address the high flow resistance issue. Rather than drawing scattered inspirations from multiple species, this study uniquely identifies a singular, highly integrated natural thermal-fluid paradigm: the hierarchical wing vein structure of the migrating dragonfly, *Pantala flavescens*. Recent biological observations reveal that the dragonfly wing not only possesses a macro-scale branching venation network for fluid transport but crucially features distinctive micro-scale V-shaped vein junctions (nested V-ribs). These V-junctions are hypothesized to passively regulate the dragonfly’s surface temperature during prolonged flight by inducing micro-vortices and disrupting local thermal boundary layers [19]. By mathematically extracting these biological V-junctions and translating them into engineering V-ribbed turbulators inside the internal cooling channels, this study proposes a unified, multi-scale bio-inspired design that inherently links macro-flow distribution with micro-turbulence generation. This disruption enhances local heat transfer without significant energy loss. Previous studies have separately reported branching cooling channels and rib turbulators for heat transfer enhancement. In contrast, the present design combines a bio-inspired branching coolant path with V-shaped ribs inside the stator cooling channel of an outer-rotor PMSM. Under the same boundary conditions, the proposed channel increases the Nusselt number by approximately 42.5%, reduces the pressure drop from nearly 20.0 kPa to approximately 4.5 kPa, and decreases the required pumping power by 77.5% compared with the helical baseline. These results indicate that the combined branching path and V-rib design can improve the thermal-fluid performance of the selected motor cooling system.

While recent advancements in biomimetic thermal management have demonstrated the efficacy of macro-scale fractal branching channels in reducing flow resistance and improving temperature uniformity [20], and micro-scale V-rib turbulators are well-documented for their ability to disrupt the boundary layer and enhance local convective heat transfer [21], the synergistic integration of these two mechanisms remains largely unexplored in the context of high-power-density electrical machines. Most prior investigations on hybrid cooling systems treat macro-channel routing and micro-surface modifications as isolated design problems. This study addresses this critical gap by proposing a coupled architecture that leverages the hydraulic efficiency of dragonfly venation and the thermal enhancement of V-ribs.

The primary advancements and key research highlights of this investigation are delineated as follows. Initially, magnetic induction levels are quantified utilizing an FE-based numerical framework for the 60 kW outer-rotor PMSM, thereby validating the feasibility of the magnetic path configuration. Furthermore, a one-way electromagnetic-to-thermal coupling method was used, which incorporates the Bertotti iron loss separation principles for precise loss estimation. Following this, the temporal evolution of iron dissipation in the stator and ohmic losses in the windings was characterized. This exploration determines the accurate heat source under rated conditions. Then, internal Bio-Inspired V-Ribbed cooling channels are proposed. This structural proposal utilizes a computational fluid dynamics (CFD) model. The proposed architecture incorporates insect wing vein bio-inspired channels. These channels reduce internal hydraulic resistance in numerical comparison. Additionally, V-shaped ribs are included to reduce local hot spots. Finally, a multi-criteria assessment of the cooling architecture’s design variables was executed to evaluate the overall performance. This evaluation uses thermal sensitivity analysis for rib height, spacing, and fin ratio. Ultimately, the heat dissipation effect and structural safety are compared. This final comparison evaluates the motor before and after optimization.

## 2. Multiphysics Modeling and Heat Source Determination

### 2.1. Prototype

This study addresses the stringent requirements for high-power density and compact spatial integration. These requirements apply to electric aviation propulsion systems and EV in-wheel drive applications. Consequently, this research designs and investigates a 60 kW class diverging from traditional inner-rotor architectures; the outer-rotor configuration of the Permanent Magnet Synchronous Motor (PMSM) represents an alternative structural paradigm. It inverts the structural layout by positioning the stator centrally and the rotor peripherally. This structural inversion inherently provides a larger air-gap radius within the same external volume envelope. Therefore, it generates a significantly higher output torque density due to the increased moment arm. However, this “heat source inside” characteristic poses a severe challenge to thermal management. The primary heat-generating stator core and windings are enclosed by the rotating rotor housing. Therefore, they lack a direct heat dissipation path to the ambient environment. Consequently, heat heavily accumulates in the stator without an advanced cooling strategy. This accumulation potentially leads to insulation degradation or permanent magnet demagnetization. To resolve this thermal contradiction, the investigated prototype adopts a specific topology [22]. This design utilizes a fractional-slot concentrated winding (FSCW) with 18 slots and 20 poles (18S/20P). The concentrated winding configuration significantly reduces the length of the end windings compared with distributed windings. Such spatial compaction effectively curtails ohmic dissipations while simultaneously decreasing the machine’s longitudinal footprint. Consequently, this configuration is ideally suited for applications with constrained axial clearance, exemplified by in-wheel drives or ducted propulsion systems. The nominal operating velocity of the machine is designated as 9000 rpm. This speed results in a fundamental frequency of up to 1500 Hz. High-frequency operation facilitates miniaturization. Nevertheless, this induces a significant escalation in both hysteretic and eddy current dissipations, positioning stator iron loss as the primary thermal contributor. Accordingly, focused thermal management is required to mitigate this dominant heat source [23].

**Figure 1:**
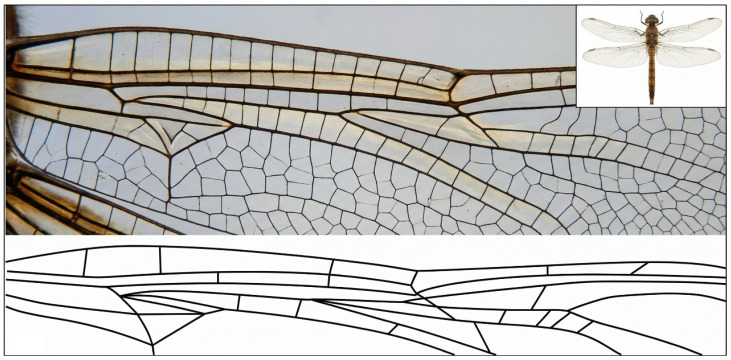
Biological inspiration: hierarchical V-shaped vein junctions extracted from the *Pantala flavescens* dragonfly wing.

To effectively manage the thermal loads under such high-frequency conditions, this paper proposes internal Bio-Inspired V-Ribbed cooling channels. As illustrated in Figure 1, the internal geometric topology is directly extracted from the wings of the migrating dragonfly, *Pantala flavescens*. Unlike conventional serpentine channels, the dragonfly wing exhibits a unique multi-scale thermal-fluid management system: a macro-scale branching venation network for global fluid distribution, coupled with micro-scale nested V-junctions (V-ribs) that passively disrupt local thermal boundary layers.

Translating this biological paradigm into the motor’s stator holder, the engineered liquid cooling heat sink features a bio-inspired branching network to minimize pumping power consumption. The translation of the biological dragonfly venation into the engineering cooling topology was quantitatively guided by Murray’s Law, a fundamental biomechanical principle that minimizes the energy required for fluid transport in branched networks. According to this biomimetic scaling law, the optimum ratio between the parent channel hydraulic diameter (*D*_0_) and the daughter channel hydraulic diameters (*D*_1_, *D*_2_) is governed by the relationship $D0^{3}=D1^{3}+D2^{3}$. To adapt this ideal biological ratio to the geometric constraints of the 60 kW PMSM stator, a spatial scaling factor was applied. This mathematical translation ensured that the maximum channel width did not exceed the stator yoke thickness, thereby avoiding magnetic saturation and structural weakening, while strictly maintaining the optimal hydraulic diameter ratio dictated by the biological model. Crucially, the inner walls of these channels are integrated with micro-scale V-shaped rib arrays, strictly mimicking the dragonfly’s V-junctions. These ribs function as turbulators, inducing periodic longitudinal vortices to effectively enhance near-wall convective heat transfer without incurring significant hydraulic resistance penalties [24].

**Figure 2:**
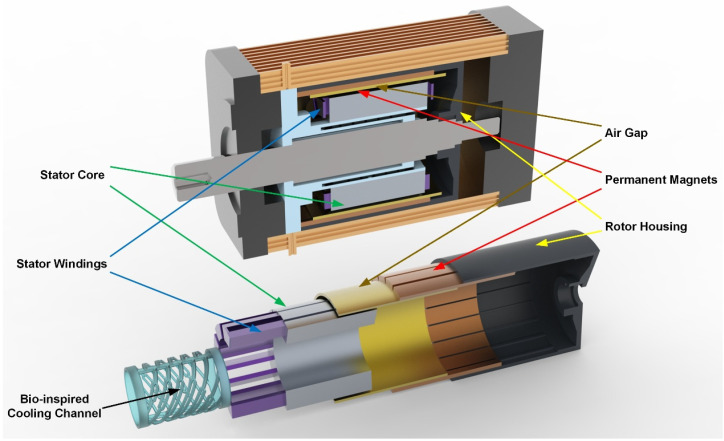
Exploded view of the proposed outer rotor PMSM with internal bio-inspired cooling channels.

The comprehensive structural configuration of the proposed high-speed outer rotor PMSM, highlighting the integration of the internal bio-inspired liquid cooling channels, is schematically illustrated in Figure 2 [25].

### 2.2. Electromagnetic Field Analysis

To accurately obtain the loss distribution data under actual operating conditions and provide high-fidelity heat source boundary conditions for subsequent thermal analysis, this section first establishes a high-precision electromagnetic field finite element (FE) numerical model. To balance computational fidelity with numerical efficiency while accounting for the axial symmetry of the machine, a 2D Time-Stepping Finite Element method (TS-FEM) is utilized for 2D transient magnetic characterization [26]. While a full 3D electromagnetic model would capture axial end-winding losses and rotor-end leakage flux more comprehensively, the 2D approach was deliberately selected. The active stator core exhibits high axial symmetry, and the 2-2DS-FEM provides a highly accurate radial distribution of the dominant heat sources (specifically, the stator iron losses) without the prohibitive computational burden associated with 3D transient magnetic coupling. It should be noted that while the electromagnetic heat source extraction is 2D, the subsequent fluid-solid conjugate heat transfer analysis is conducted in a fully 3D environment, where critical 3D thermal effects, such as end-winding ventilation cooling, are mathematically compensated for using equivalent heat transfer coefficients. Within the electromagnetic solver’s domain, the magnetic vector potential A is defined as the governing state variable to delineate the magnetic field profiles. Neglecting the displacement current, the governing equations for the 2D transient electromagnetic field derived from Maxwell’s equations account for the source current density and induced eddy current density, incorporating the nonlinear magnetic reluctivity and electrical conductivity of the ferromagnetic materials. The simulation model is constructed on the co-simulation platform of Motor-CAD. The stator core utilizes 35W270 high-grade silicon steel sheets, a material characterized by low specific iron loss, to effectively mitigate the surge in iron loss during high-frequency operation [27]. The permanent magnets are composed of N38UH grade NdFeB material with radial magnetization, ensuring sufficient resistance to demagnetization at elevated temperatures [28]. Dirichlet boundary conditions are applied to the outermost boundary of the solution domain, setting the magnetic vector potential to zero to simulate the attenuation of the magnetic field at infinity.

**Figure 3:**
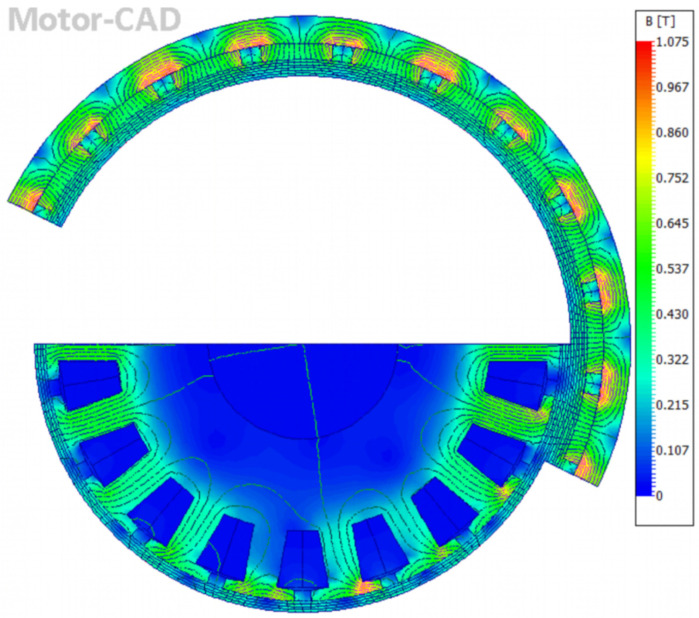
Magnetic flux density contour and flux line distribution under rated load conditions.

Utilizing the previously described FE model, a comprehensive investigation into the magnetic field distribution under nominal load was conducted, with the resulting flux density contours and flux line patterns illustrated in Figure 3. As observed, the magnetic flux lines exhibit high continuity and follow well-defined paths through the stator teeth, yoke, and air gap, consistent with the initial design objectives. Notably, the absence of significant local magnetic saturation validates the appropriateness of the magnetic circuit configuration. Moreover, a quantitative assessment of the air-gap magnetic flux density reveals a peak value of approximately 1.037 T under nominal operating conditions. This value reflects a design trade-off: it ensures that the motor possesses a sufficiently high air-gap magnetic loading to output high torque density while retaining an appropriate safety margin to prevent the stator tooth tips from entering deep saturation under overload conditions, which could degrade control performance. Furthermore, Fast Fourier Transform (FFT) analysis of the no-load back-EMF waveform reveals excellent sinusoidal fidelity with a low Total Harmonic Distortion (THD). This is of critical significance for reducing high-frequency harmonic losses, suppressing torque ripple, and minimizing electromagnetic vibration and noise during motor operation.

To examine the numerical consistency of the established 2D TS-FEM electromagnetic model, the predicted electromagnetic performance was benchmarked against fundamental analytical magnetic circuit calculations. Specifically, the fundamental amplitude of the no-load phase back electromotive force (back-EMF) obtained from the TS-FEM was compared with the analytical result derived from the classical equivalent magnetic circuit method, considering the fractional-slot concentrated winding topology and the permanent magnet remanence. The comparison showed a deviation of 3.5%, indicating reasonable agreement between the numerical and analytical results. It should be noted that this comparison provides an analytical consistency check only, while full experimental validation remains necessary in future prototype testing.

### 2.3. Loss Calculation Results

At a nominal operational velocity of 9000 rpm, the machine functions at a fundamental electrical frequency of 1500 Hz. At such high frequencies, the loss characteristics differ significantly from conventional low-speed machines, making the accurate separation and calculation of loss components critical for thermal source mapping. In this study, the losses are calculated using the 2D transient finite element method combined with the Bertotti iron loss separation model, ensuring that the effects of high-order harmonics on the core are fully captured.

Adopting the Bertotti iron loss separation framework, the aggregate core dissipation (P_Fe_) is partitioned into three constituent components: hysteresis loss (P_h_), classical eddy current loss (P_c_), and anomalous excess loss (P_e_). The mathematical formulation is given by:

(1) $$P_{Fe}=P_{h}+P_{c}+P_{e}=K_{h}fB_{m}^{\alpha }+K_{c}f^{2}B_{m}^{2}+K_{e}f^{1.5}B_{m}^{1.5}$$

In this formulation, f denotes the magnetic field excitation frequency, and B_m_ represents the peak magnetic flux density. The parameters $K_{h}$, $K_{c}$, and $K_{e}$ correspond to the hysteresis, classical eddy current, and excess loss coefficients, respectively. All of these are characteristic constants of the 35W270 silicon steel grade. Specifically, the fitted empirical values utilized in this study are $K_{h}=165$, $K_{c}=0.38$, and $K_{e}=2.1$. Furthermore, α signifies the Steinmetz exponent, which is set to 2.0. Following the transient electromagnetic solution, a unidirectional coupling strategy is employed to map the 2D losses into the 3D thermal model. The cycle-averaged transient electromagnetic losses, including both the core losses and winding copper losses, are extracted as discrete spatial nodal loss densities. These 2D cross-sectional heat distributions are subsequently extruded along the axial length of the active motor components and interpolated onto the 3D computational fluid dynamics mesh. This mapping procedure transforms the electromagnetic losses into steady-state volumetric heat generation rates, ensuring that localized high-frequency harmonic loss concentrations are faithfully represented in the ultimate 3D thermal evaluation.

Based on this analytical model coupled with TS-FEM, the detailed loss breakdown of the motor under rated load is obtained and summarized in Table 1. The 174 W total loss in Table 1 requires clarification. This value strictly represents the isolated electromagnetic losses under rated conditions. A high-speed motor at 9000 rpm generates significant mechanical losses, and these include windage loss and bearing friction. These mechanical losses can significantly increase the overall motor temperature.

However, this study focuses on the internal cooling channels, and it evaluates their heat transfer performance. Mechanical windage losses primarily affect the external rotor and the air gap. They do not directly impact the internal stator core. The proposed bio-inspired cooling jacket is located inside the stator core, so the current thermal model deliberately excludes these mechanical parameters.

This simplification establishes a fair comparative baseline. It ensures a strict single-variable experiment. Thus, the observed thermal differences solely reflect the cooling efficacy of the internal channels.

The analysis of the loss constituents highlights that the stator iron loss is the absolute dominant heat source. As evidenced by the data in Table 1, the total stator iron loss reaches 132.9 W, which accounts for approximately 76.4% of the total electromagnetic loss. Such prevalence is a hallmark of high-speed machine performance, where the hysteretic and eddy current dissipations in the core laminations exhibit a quadratic dependency on the operational frequency.

Detailed spatial partitioning of the core dissipations highlights a pronounced heterogeneity; specifically, the loss localized within the stator teeth reaches 111.7 W, markedly surpassing the 21.16 W quantified in the stator yoke. This disparity arises because the stator teeth are subjected to higher magnetic flux densities and more intense harmonic flux modulations than the yoke. Therefore, the stator teeth are identified as the most critical “hot spots” in the thermal model, necessitating a cooling path that can effectively extract heat from the tooth tips through the yoke to the housing.

In contrast to the iron loss, the winding copper loss is effectively suppressed. Thanks to the optimized slot fill factor of 0.5 and the selection of a 1.707 mm conductor diameter, the DC copper loss under rated current is calculated to be 38 W. This value constitutes only 21.8% of the total loss. The relatively low copper loss implies that the thermal load density within the slots is manageable, and the winding temperature rise will be primarily driven by the heat conduction from the adjacent high-loss stator teeth rather than by self-heating. This provides a substantial safety margin for short-term overload operations where ohmic losses would typically scale quadratically with current.

Regarding the rotor side, the simulation results confirm the thermal advantages of the proposed design. The eddy current dissipations within the permanent magnets are quantified at a minimal 2.224 W, whereas the rotor core loss is virtually negligible, measuring approximately 0.92 W. The combined rotor-side loss is less than 3.2 W, representing less than 2% of the total loss. This minimal heat generation confirms that the rotor operates in a thermally “cold” state relative to the stator. Consequently, the risk of irreversible demagnetization due to rotor self-heating is minimal, and the cooling design can be simplified by focusing almost exclusively on the stator heat extraction without implementing complex active rotor cooling mechanisms.

Mechanical losses, including windage loss and bearing friction loss, were not included as heat sources in the final thermal simulation. They were discussed qualitatively as possible additional heat sources in practical operation. Therefore, the final temperature results should be interpreted as numerical predictions based on electromagnetic heat sources only.

## 3. Comparative Analysis of Thermal Characteristics

### 3.1. Heat Conduction Equations

When formulating the thermal analysis model, several justified simplifying assumptions are incorporated. First, the skin effect is deemed negligible. At the fundamental frequency of 1500 Hz, the theoretical skin depth of copper is approximately 1.68 mm, which is significantly larger than the radius of the selected winding conductor (1.707 mm diameter). Thus, high-frequency AC resistance increases are minimal. Second, thermal radiation is excluded. Because the proposed bio-inspired water jacket effectively suppresses the peak internal temperatures below 60 °C (approximately 333 K), radiative heat flux remains orders of magnitude smaller than forced convective heat transfer. Consequently, the simulation exclusively accounts for heat conduction and convective transport mechanisms. Furthermore, explicitly resolving the complex high-speed rotational fluid dynamics, such as Taylor Couette flow, within the air gap at 9000 rpm entails prohibitive computational costs. To balance computational efficiency and thermal fidelity, this highly turbulent aerodynamic agitation is modeled using an empirically validated equivalent thermal conductivity approach. Grounded in heat transfer theory, the governing three-dimensional heat conduction differential equations for the steady state thermal behavior of the motor components are formulated as follows. Under steady state conditions, the temperature field does not change with time, thus $\frac{\partial T}{\partial t}=0$.

(2) $$q+k_{x}\frac{\partial^{2}T}{\partial x^{2}}+k_{y}\frac{\partial^{2}T}{\partial y^{2}}+k_{z}\frac{\partial^{2}T}{\partial z^{2}}=c\rho \frac{\partial T}{\partial t}$$

where $k_{x,y,z}$ denotes the thermal conductivity of the motor, T denotes temperature, q denotes the heat generation rate, c denotes specific heat capacity, and ρ denotes density.

In the motor’s thermal field simulation, the energy equilibrium condition at the fluid-solid interaction (FSI) interface is formulated as:

(3) $$-\lambda_{i}\left(\frac{\partial T}{\partial n}\right)=\alpha_{i}\left(T-T_{f}\right)$$

where n is the normal vector of the boundary surface, $\alpha_{i}$ is the heat transfer coefficient boundary surface, and $T_{f}$ is the temperature of the cooling medium.

It should be emphasized that for the thermal field analysis, the air gap separating the stator and rotor is simplified as a stationary, solid cylindrical shell. However, this simplification inherently neglects the complex fluid dynamic effects induced by rotor rotation. To compensate for this discrepancy and ensure the equivalence of heat transfer accuracy, an equivalent thermal conductivity is introduced [29]. This parameter analytically correlates the convective heat transport mechanisms inherent in the fluid motion with the conductive properties of the stationary solid domain. Thus, it ensures that the thermal performance of the idealized model remains congruent with the actual fluid-dynamic environment. The resultant equivalent thermal conductivity for the air-gap region is determined via the following expression:

(4) $$\lambda_{a}=0.0019{\left(\frac{D_{2}}{D_{1}}\right)}^{-2.9084}{\left(\frac{w_{r}Re\delta }{60v_{r}}\right)}^{0.4614\ln\left(3.33361-\left(\frac{D_{2}}{D_{1}}\right)\right)}$$

where $\delta =D_{1}-D_{2}$, δ is the air-gap length. Aerodynamic agitation induced by the rotor’s rotation intensifies the air turbulence across the stator surfaces, thereby significantly enhancing the convective heat transfer and thermal dissipation of the end windings. Hence, the convection heat transfer coefficient of the stator end surface is described as follows:

(5) $$\alpha_{1}=9.73+14{v_{t}}^{0.62}$$

where $v_{t}$ is the wind speed of the stator end surface. The rotor can be simplified as the natural cooling, and the surface convection heat transfer coefficient of the rotor shell can be expressed as follows:

(6) $$\alpha_{2}=14\sqrt[{3}]{\frac{T_{c}}{25}}$$

where $T_{c}$ denotes the external ambient temperature. The cooling medium primarily dissipates the heat generated by the PM motor during operation. In computational analysis, the coolant is typically treated as an incompressible fluid. Moreover, the standard k-ε model is adopted to account for its turbulent behavior [30], and the cooling medium velocity is continuous under steady flow. Additionally, the transformation between momentum and energy can be formulated as:

(7) $$\left\{\begin{array}{c} \nabla v=0\\ \rho_{f}\frac{dv}{dt}=-\nabla p+\mu \nabla^{2}v\\ \nabla \left(\lambda_{f}\nabla T_{f}\right)=\rho_{f}C_{f}\frac{\partial T_{f}}{\partial t}+\nabla \left(\rho_{f}C_{f}vT_{f}\right) \end{array}\right.$$

where v, μ, and p are the fluid velocity, dynamic viscosity, and pressure of the cooling medium, respectively, $\rho_{f}$, $C_{f}$ and $\lambda_{f}$ are the density, specific heat capacity, and thermal conductivity of the cooling medium, respectively. It should be noted that for the evaluation of the motor’s ultimate continuous cooling performance, a steady-state fluid-solid coupled computational fluid dynamics simulation is conducted. Therefore, all time derivative terms in the aforementioned governing equations are set to zero in the numerical solver. It is acknowledged that the complex biologically inspired branching networks and micro-scale V-ribs inherently induce secondary flows and localized recirculation. While advanced models such as the Shear Stress Transport k-omega model are specifically tailored for strong streamline curvatures and near-wall thermal boundary layers, they impose high computational costs and convergence difficulties during extensive parametric analyses. To strictly justify the employment of the standard model, a cross-validation was performed. The optimized bio-inspired channel under rated conditions was independently simulated using the highly robust Shear Stress Transport model. The comparison revealed highly consistent macroscopic thermal-hydraulic performance. Specifically, the maximum stator temperature predicted by the Shear Stress Transport k-omega model was 48.3 degrees Celsius, compared to the 48.0 degrees Celsius predicted by the standard model, representing a minimal relative deviation of less than 0.7 percent. Furthermore, the total fluid pressure drop exhibited a slight deviation of approximately 3.5 percent. Therefore, considering that the discrepancies in the predicted macroscopic thermal profiles and global pressure drops between the two models are within an acceptable 5 percent engineering margin, the standard k-epsilon model was considered suitable for the comparative thermal-fluid analysis in this study. The heat transfer coefficient on the winding end surface affects the thermal behavior and operating efficiency of the motor. On the basis of fluid similarity principles, the convection heat transfer coefficient of the flow channel can be derived from the following equation [31]:

(8) $$\left\{\begin{array}{c} Re_{r}=\frac{vD_{w}}{v_{f}}\\ p_{r}=\frac{\mu C_{f}}{\lambda_{f}}\\ Nu_{r}=0.023R{e_{r}}^{0.8}{P_{r}}^{0.4}\\ h_{f}=\frac{Nu_{r}\lambda_{f}}{D_{w}} \end{array}\right.$$

For the non-circular cooling channels, the hydraulic diameter is defined as Dh = 4Ac/P, where Ac is the cross-sectional flow area and P is the wetted perimeter.

The Reynolds number, Prandtl number, and Nusselt number are defined as Re = ρvDh/μ, Pr = μcp/λ, and Nu = hDh/λ, respectively. The reference Nusselt number for turbulent flow in a smooth channel is calculated using the Dittus-Boelter correlation, $Nur=0.023Re^{0.8}Pr^{0.4}$, where ρ is the coolant density, v is the mean flow velocity, μ is the dynamic viscosity, cp is the specific heat capacity, λ is the thermal conductivity, and h is the convective heat-transfer coefficient.

### 3.2. Geometric Models and Bio-Inspired Channel Design

**Figure 4:**
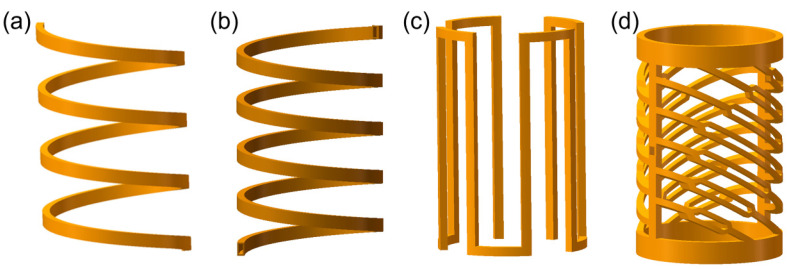
Geometric model of four cooling channels. (**a**) Helical channel, (**b**) helical ribbed channel, (**c**) axial channel, and (**d**) bio-inspired channel.

The thermal dissipation capacity of the rotor constitutes a pivotal determinant of the machine’s overall operational integrity and performance. To evaluate the thermal dissipation performance under different channel structures, four distinct cooling channel configurations are integrated into the motor housing, as shown in Figure 4. Traditional channels include the axial channel, helical channel, and helical ribbed channel. The axial channel features straight paths parallel to the motor shaft, offering low manufacturing complexity but often resulting in uneven temperature distribution. The helical channel extends the coolant path to increase the heat dissipation area. The helical ribbed channel introduces V-shaped grooves to enhance turbulence and disrupt the boundary layer, thereby improving heat dissipation performance. It is worth noting that the conventional serpentine (Z-type) channel, although common in traditional machines, is explicitly excluded from this comparative study. A serpentine topology requires complex 180-degree U-bends at the end regions, which significantly increases the axial length of the internal water jacket. This contradicts the stringent axial space constraints of the targeted in-wheel and electric aviation applications. Therefore, the axial channel, helical channel, and helical ribbed channel are selected as the most realistic and comparable conventional baselines for this compact outer-rotor topology. Unlike traditional periodic structures, the bio-inspired channel is inspired by the dragonfly wing venation of *Pantala flavescens*. This design employs a hierarchical branching network that optimizes the fluid distribution across the stator surface [32]. Through the optimization of bifurcation angles and the cross-sectional area of the secondary channels, the bio-inspired architecture mitigates the thermal stagnation zones inherent in conventional helical configurations. By emulating the low-dissipation transport networks observed in dragonfly wing venation, this design significantly curtails hydraulic resistance. To facilitate an equitable evaluation, the core geometric specifications of the stator and the baseline cooling parameters are kept constant. The detailed dimensional attributes of the cooling channels are tabulated in Table 2. To clearly articulate the design translation from the dragonfly wing pattern to the final cooling geometry, the dragonfly wing venation was mathematically abstracted and governed by Murray’s Law to ascertain the optimal branching diameter ratios. Unlike conventional helical or simply branched channels that frequently suffer from severe pressure drops and localized thermal bottlenecks, this biomimetic translation inherently balances the fluid distribution. The abstracted macroscopic network was subsequently scaled to strictly fit the radial spatial constraints of the stator yoke, ensuring both structural rigidity and magnetic flux integrity. What truly distinguishes this geometry from existing heat exchanger designs is its multi-scale synergistic architecture. It combines the macroscopic bio-inspired branching network, which optimizes global hydraulic transport, with the microscopic V-rib arrays, which are specifically embedded to disrupt the near-wall thermal boundary layer at high-temperature locations.

To successfully adapt the bio-inspired dragonfly-vein concept into the PMSM cooling system, a systematic geometric scaling and optimization procedure was implemented. Unlike unconstrained biological growth, the biomimetic channel must adhere to the strict spatial limitations of the stator yoke. Therefore, the maximum width and depth of the primary channels were rigidly constrained to preserve the structural integrity of the stator and to ensure sufficient magnetic flux paths, thereby preventing localized magnetic saturation. Concurrently, the microscopic V-rib arrays embedded within the channels underwent a rigorous parametric optimization. As detailed in the subsequent analysis, parameters such as the rib inclination angle were systematically varied to determine the optimal configuration (45°), achieving the most favorable trade-off between the convective heat transfer enhancement and the inevitably induced hydraulic penalty.

### 3.3. Mesh Generation and Boundary Conditions

**Figure 5:**
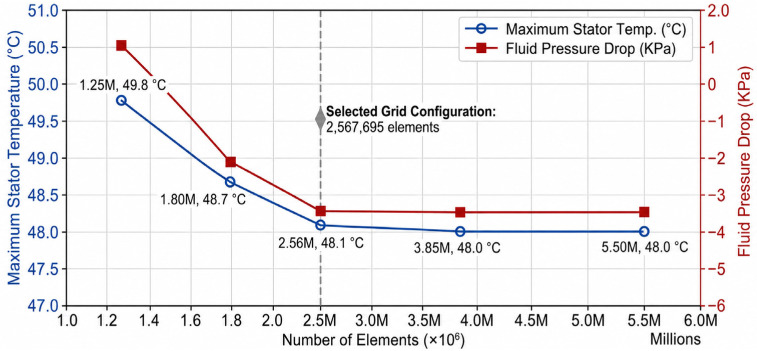
Grid independence validation data for the bio-inspired channel model under rated operating conditions.

**Figure 6:**
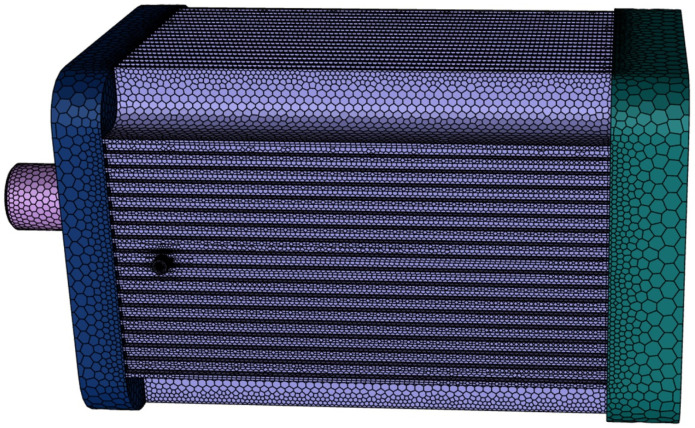
Finite element model of the motor.

The fidelity of the thermal field prediction is critically contingent upon the grid discretization quality, with particular sensitivity observed at the intricate branching nodes of the bio-inspired manifold. The regular stator and rotor components are discretized using structured hexahedral elements, while the intricate cooling channels are modeled with a tetrahedral mesh to capture the near-wall turbulence effects. The cooling medium used in all numerical simulations was pure water with a constant inlet temperature of 20 °C. The coolant properties were defined as a density of 998.2 kg/m^3^, a specific heat capacity of 4182 J/(kg·K), a thermal conductivity of 0.6 W/(m·K), and a dynamic viscosity of 1.0 × 10^−3^ kg/(m·s). To guarantee the numerical accuracy of the temperature and pressure predictions and to optimize the computational cost, a rigorous mesh independence study was conducted prior to the primary simulations. Five different mesh densities ranging from approximately 1.2 million to 5.5 million elements were evaluated for the bio-inspired channel model under rated operating conditions. Both the maximum stator temperature and the fluid pressure drop were selected as the primary monitoring parameters. The evaluation indicated that as the grid resolution increased from 1.2 million to 2.56 million elements, the predicted maximum temperature and pressure converged significantly, as clearly depicted in Figure 5. When the mesh density was further refined from 2.56 million to 3.85 million and ultimately to 5.5 million elements, the relative variation in the maximum stator temperature was less than 0.2 percent, and the variation in the fluid pressure drop was less than 1.0 percent. Because further refinement incurs a substantially higher computational burden without providing meaningful gains in precision, the grid configuration with 2,567,695 elements was selected as the optimized and reliable baseline for all subsequent multiphysics simulations. The representative finite element architecture of the machine is illustrated in Figure 6. To ensure full reproducibility and to guarantee the numerical accuracy of the predicted cooling performance, the comprehensive computational fluid dynamics and thermal model settings are explicitly detailed. The same coolant inlet temperature and thermophysical properties were used for all cooling-channel cases. Numerical boundary constraints are established to replicate the actual operational environment of the motor. The boundary conditions consist of a velocity inlet ranging from 0.5 to 2.0 m per second, a pressure outlet at 0 pascals gauge, and coupled no-slip fluid-solid interfaces. The standard k epsilon turbulence model, combined with Enhanced Wall Treatment maintaining a y plus value below 5 in critical heat transfer regions, was selected to accurately capture the near-wall convective heat transfer without incurring the prohibitive computational costs of resolving the viscous sublayer directly. A pressure-based steady state solver utilizing the SIMPLEC algorithm and second-order upwind spatial discretization was applied for momentum, turbulent kinetic energy, dissipation rate, and energy. The simulations were deemed rigorously converged only when the energy residual dropped below 10^–6^, and the continuity and momentum residuals dropped below 10^–4^. Furthermore, global energy balance checks confirmed that the net mass flux was below 10^–5^ kg per second and the discrepancy between the total volumetric heat generated and the net heat removed by the coolant was less than 1 percent. Finally, the heat transfer between the fluid and the channel wall is governed by the coupled thermal boundary condition, ensuring energy conservation across the interface:

(9) $$k_{s}\left(\frac{\partial T_{s}}{\partial n}\right)=k_{f}\left(\frac{\partial T_{f}}{\partial n}\right)$$

where the subscripts s and f denote the solid and fluid domains, respectively.

### 3.4. Numerical Methodology Validation

To ensure the accuracy and reliability of the computational fluid dynamics (CFD) methodology employed in this study, a validation study was conducted prior to simulating the complex bio-inspired topologies. Given that the physical prototypes of the highly intricate bio-inspired micro-V-ribs are currently in the manufacturing phase, the validation focused on benchmarking the numerical framework against published experimental data. Specifically, a baseline straight channel integrated with rib turbulators, identical in scale and flow conditions to the experimental study conducted by Tamang et al. [24], was numerically reconstructed. The identical meshing strategy and turbulence modeling approach used for the proposed bio-inspired motor cooling channels were applied. The numerically predicted Nusselt numbers and friction factors were extracted and compared with the experimental measurements from [24]. The results demonstrate that the maximum deviation between the CFD predictions and the experimental data remains within 6.8% for the Nusselt number and 7.2% for the friction factor. These agreements support the use of the selected numerical scheme to capture the secondary vortex flows and heat transfer enhancement induced by the ribs for the following comparative analysis.

## 4. Results and Discussion

### 4.1. Comparative Analysis of Fluid Field Characteristics

**Figure 7:**
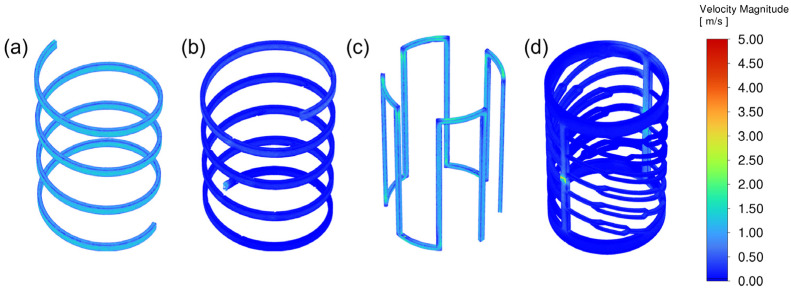
Velocity magnitude distribution of the motor under different coolant channels. (**a**) Helical channel, (**b**) helical ribbed channel, (**c**) axial channel, and (**d**) bio-inspired channel.

**Figure 8:**
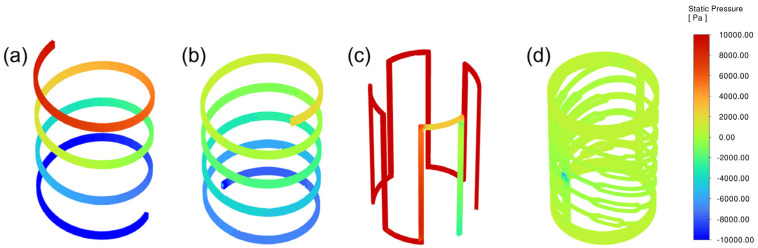
Static pressure distribution of the motor under different coolant channels. (**a**) Helical channel, (**b**) helical ribbed channel, (**c**) axial channel, and (**d**) bio-inspired channel.

The hydraulic behavior of the coolant serves as the physical precursor to the thermal response. As illustrated by the velocity streamlines and pressure contours in Figure 7 and Figure 8 for each topology, each channel design exhibits distinct flow behavior under the rated inlet velocity. Figure 7a shows that the helical channel presents a flow pattern with non-uniform velocity magnitudes. Velocity magnitudes mainly concentrate in low ranges of 0.5–2.0 m/s. Meanwhile, Figure 8a shows a pronounced static pressure gradient. High pressure accumulates at the red/orange regions of the inlet section, while significant pressure drops occur at the outlet, forming stagnation tendencies that restrict convective heat transfer efficiency. The helical ribbed channel maintains low velocity levels, predominantly below 1.0 m/s, as shown in Figure 7b. Additionally, compared with the helical channel, the helical ribbed channel shows milder pressure variation, yet it still exhibits pressure attenuation along the flow path. In contrast, the axial channel shows more severe high-pressure areas, as shown in Figure 8c. Meanwhile, it is found from Figure 7c that the axial channel generates localized high-velocity zones at branching junctions to disrupt laminar stagnation. The bio-inspired channel shows a more uniform pressure distribution than the selected baseline channels, as shown in Figure 7d and Figure 8d. The pressure drop of the bio-inspired channel is approximately 4.5 kPa, which is much lower than the nearly 20.0 kPa pressure drop of the helical channel under the same inlet condition. This lower pressure drop reduces the required pumping power by 77.5%. The velocity contours also indicate reduced stagnation regions in the bio-inspired channel.

The bio-inspired channel shows improved thermal-fluid performance compared with the selected baseline channels. Thermally, the inclusion of micro-V-rib arrays changes the near-wall flow and increases local mixing. This effect disturbs the thermal boundary layer and increases the Nusselt number by approximately 42.5% compared with the helical baseline channel. From a hydraulic perspective, the branching channel mitigates severe pressure accumulation. The pressure drop of the bio-inspired channel is approximately 4.5 kPa, while the helical channel shows a pressure drop of nearly 20.0 kPa under the same inlet condition. Consequently, the required pumping power of the bio-inspired channel is decreased by 77.5%. These quantitative results support the thermal-fluid advantage of the proposed bio-inspired configuration in the numerical comparison.

### 4.2. Thermal Field Distribution and Component Temperatures

**Figure 9:**
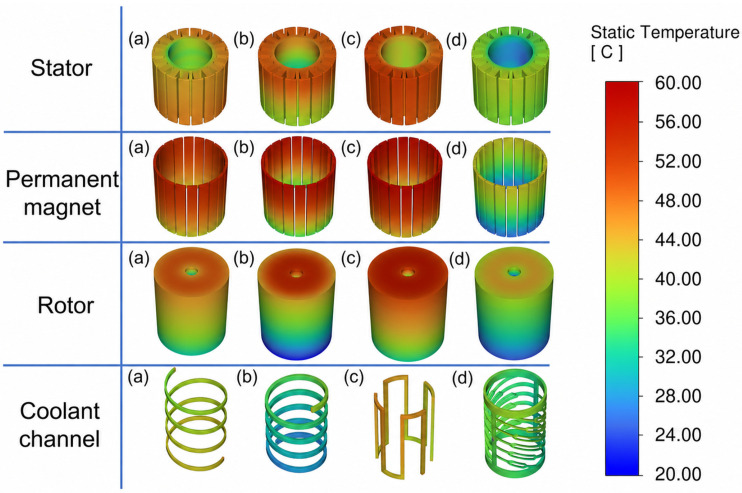
The temperature fields of different components of the motor. (**a**) Helical channel, (**b**) helical ribbed channel, (**c**) axial channel, and (**d**) bio-inspired channel.

Based on the simulation results of the steady-state temperature fields shown in Figure 9, the cooling performance of the four investigated channel topologies under rated load varies significantly. In the temperature contours for the stator and windings, structures (a) helical channel, (b) helical ribbed channel, and (c) axial channel exhibit pronounced thermal saturation, with large surface areas approaching the red zone. The temperatures of most regions are maintained at around 60 °C. In contrast, the bio-inspired structure (d) shows lower temperatures in the numerical comparison. Specifically, the stator surface of structure (d) is primarily dominated by cyan and yellow hues, with temperatures in most regions maintained between 40 °C and 48 °C. The temperature fields of the bio-inspired structure for the stator have a substantial reduction compared to other structures. The temperature reduction in the bio-inspired design is also observed in the permanent magnet section, where the risk of irreversible demagnetization is highest. As illustrated in the second row of Figure 9, the permanent magnets of traditional channels (a), (b), and (c) are almost entirely in a high-temperature state near 60 °C. Conversely, the permanent magnets of the bio-inspired structure (d) exhibit a clear temperature gradient. The bottom and middle regions appear in deep blue (approximately 42 °C), reducing the risk of localized overheating. These numerical results indicate that the intricate branching of the bio-inspired channel improves convective heat transfer within the core winding regions.

Regarding the rotor components, which are critical for operational reliability, structure (d) shows a maximum temperature of approximately 57 °C in the numerical model. This result suggests a lower high-temperature risk than the over 65 °C case observed in structure (c). The cooling-channel contours indicate that the bio-inspired structure (d) provides broader coverage and a more uniform flow distribution. This non-uniform topology allows the cooling medium to reach areas with high heat flux. Overall, the bio-inspired channel structure (d) reduces the peak temperatures of the motor components and lowers temperature gradients in the numerical comparison. To support a clearer hydraulic analysis, the theoretical pumping power was calculated as the product of the total pressure drop and the volumetric flow rate. Under the rated operating condition, the inlet velocity was set to 1.5 m/s. The effective inlet flow area was 2.0 × 10^−4^ m^2^, giving a volumetric flow rate of 3.0 × 10^−4^ m^3^/s according to $Q=v_{in}A_{in}$. The hydraulic pumping power was then calculated by $P_{pump}=\Delta pQ$, where Δp is the pressure drop between the inlet and outlet, and Q is the volumetric flow rate. Based on the pressure drop values of 4.5 kPa and 20.0 kPa, the pumping powers of the bio-inspired channel and helical baseline are 1.35 W and 6.0 W, respectively. These values indicate that the bio-inspired channel reduces pumping power by 77.5% under the same inlet condition. In conclusion, the bio-inspired channel structure is employed in the following discussions to analyze additional factors influencing the heat dissipation performance of the motor.

### 4.3. Influence of Rib Inclination Angle on Heat Dissipation Performance

**Figure 10:**
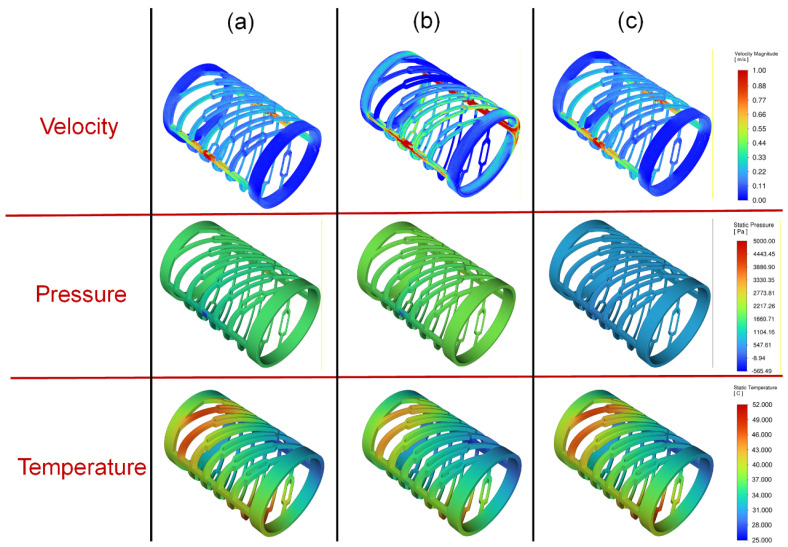
The comparative simulation results for the coolant channels under different rib inclination angles. (**a**) 30°. (**b**) 45°. (**c**) 60°.

Figure 10 presents the comparative contours of velocity, static temperature, and static pressure for the coolant channels evaluated at rib inclination angles of 30°, 45°, and 60°. The numerical results show that the 45° inclination angle gives the most favorable heat dissipation performance among the tested cases. As demonstrated in the velocity contours, increasing the angle from 30° to 45° guides the coolant into the circumferential branch channels, promoting higher local flow velocities and enhancing fluid mixing, which are highly conducive to convective heat dissipation. This change is reflected in the temperature field. The 45° configuration shows a more uniform temperature distribution and a smaller localized high-temperature zone near the outlet than the 30° case. While the 60° configuration also improves upon the 30° baseline, it still retains a slightly larger maximum temperature area compared to the 45° design, indicating a relative decline in heat transfer efficiency. From a hydraulic perspective, the static pressure contours indicate that although the overall pressure drop generally decreases as the angle approaches 60°, the 45° configuration maintains a moderate and acceptable pressure penalty while maintaining lower temperatures in the numerical comparison. Consequently, considering the critical balance between maximizing heat dissipation and controlling flow resistance, the 45° rib inclination angle is selected as the favorable geometric parameter for the cooling structure among the tested cases.

### 4.4. Influence of Inlet Velocity on Heat Dissipation Performance

**Figure 11:**
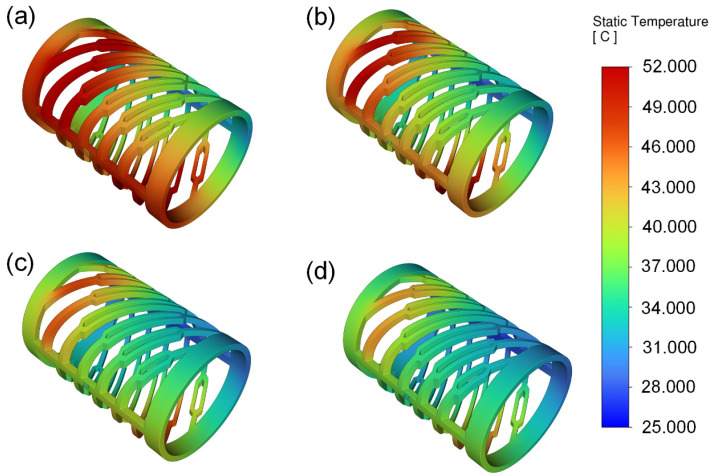
The temperature fields of the motor under different inlet velocities, (**a**) 0.5 m/s, (**b**) 1m/s, (**c**) 1.5 m/s, and (**d**) 2.0 m/s.

The heat dissipation performance of the proposed bio-inspired channel is further analyzed through a sensitivity analysis across a spectrum of inlet velocities [33], as depicted in Figure 11. As the coolant flow rate increases, the maximum temperatures of the channel walls and the fluid decrease continuously. At low flow rates, the non-uniform bio-inspired topology triggers small disturbances in the fluid. These disturbances effectively break the thermal boundary layer commonly found in traditional straight channels. Consequently, heat transfers rapidly from the high-temperature walls to the core of the cooling medium. This indicates a clear initial heat exchange effect in the numerical model. Higher flow velocities further enhance the convective heat transfer effect. This process reduces peak temperatures and smooths the temperature gradient along the channel in the numerical results. Temperature contours show that the unique bio-inspired geometry reduces flow dead zones in the numerical results. This design reduces local hot spots in numerical results. Therefore, it reduces the risk of thermal stress concentration within the motor. The bio-inspired structure maintains a low temperature profile across a wide range of flow rates. It suggests that the motor can operate within a lower-temperature range under the conditions studied. These results provide a numerical basis for the thermal safety design of high-power-density motors.

## 5. Conclusions

This study conducted a comprehensive multiphysics investigation. We addressed the thermal bottlenecks of outer rotor motors. A one-way electromagnetic-to-thermal coupling model was established. This model calculated the heat sources of a 60 kW prototype. Results identified stator iron loss as the dominant heat source. It accounted for 76.4% of the total electromagnetic loss. Conversely, the rotor-side loss remained negligible. This confirmed the necessity of targeted stator thermal intervention.

This paper proposed internal bio-inspired channels with hierarchical V-ribs extracted from the dragonfly wing. Comparative CFD results indicate that the bio-inspired design shows lower component temperatures than the selected baseline channels under the same numerical boundary conditions. The proposed channels reduce severe fluid stagnation regions and decrease the maximum stator and permanent magnet temperatures to 48 °C and 42 °C in the numerical model. Furthermore, the rib inclination angle significantly influences the cooling performance. The 45° angle gives the most favorable overall heat dissipation among the tested angles. It balances convective heat transfer and hydraulic pressure penalty in numerical comparison.

Mechanical losses were not included in the final thermal simulation. Therefore, the reported temperatures represent numerical predictions under electromagnetic heat-source conditions. In practical high-speed operation, windage loss and bearing friction may further increase the absolute temperature level.

Despite these numerical results, this study has several limitations. First, the current thermal model deliberately excludes mechanical losses, such as windage and bearing friction, to strictly isolate the internal cooling efficacy of the proposed channel topologies. At an elevated speed of 9000 revolutions per minute, aerodynamic windage becomes a prominent heat source. This assumption may lead to lower predicted temperatures than those in practical operation, especially near the air gap, rotor surface, and end shields. However, from a heat transfer perspective, the primary heat source relies on outward radial conduction to the cooling jacket. Because the air gap acts as a high thermal resistance barrier, introducing windage losses would primarily elevate the global temperature baseline without fundamentally altering the temperature gradients across the stator jacket interface. Second, the thermal model assumes perfect thermal contact at the solid interfaces, specifically between the stator, housing, and cooling jacket, neglecting the interface thermal resistance inherent in practical assembly. This idealization is applied uniformly across all simulated models. Therefore, the reported temperatures may be lower than the actual temperatures in practical motor assemblies. Consequently, omitting mechanical losses and contact resistances may affect the absolute temperature values, but it does not change the relative comparison among channel topologies under the same assumptions. Third, the current investigation into geometric parameters, such as the V-rib inclination angle, is fundamentally a discrete parametric sensitivity analysis. Because formal mathematical optimization techniques, such as Response Surface Methodology or multi-objective optimization, were not employed, the identified parameters represent highly favorable configurations rather than absolute global optimums. Fourth, parametric evaluation is derived from deterministic steady-state computational fluid dynamics simulations. While steady state analysis effectively evaluates the worst-case ultimate thermal equilibrium of the motor, it does not fully capture the dynamic thermal responses, localized hot spot migrations, or structural fatigue induced by transient duty cycles and thermal cycling. Finally, formal uncertainty quantification regarding manufacturing tolerances, specifically the surface roughness and geometric deviations of the micro-scale V-ribs, has not yet been explicitly modeled. It should be noted that the present conclusions are based on numerical simulations rather than experimental measurements. Experimental validation of the coupled electromagnetic and thermal model, together with the fluid dynamic predictions, has not yet been conducted in this study. Manufacturing such complex internal microstructures presents practical challenges that will likely necessitate high-precision metal additive manufacturing. Future research will focus on constructing physical prototypes to measure the actual surface roughness and further validate the numerical models. Furthermore, because prolonged thermal cycling inherently causes structural fatigue, subsequent studies will perform comprehensive thermomechanical stress analyses.

## Figures and Tables

**Table 1:** Electromagnetic loss breakdown of the motor under rated conditions.

Region	Loss Component	Value (W)	Proportion (%)
Stator	Teeth Iron Loss	111.70	64.2
	Yoke Iron Loss	21.16	12.2
	Winding Copper Loss	38.00	21.8
Rotor	PM Eddy Current Loss	2.22	1.3
	Stator Core Loss	0.92	0.5
Overall	Total Electromagnetic Loss	174.00	100.0

**Table 2:** Geometric parameters of the four cooling channels.

Channel Type	Hydraulic Diameter	Channel Height
Axial channel	3.5 mm	75 mm
Helical channel	3.5 mm	75 mm
Helical ribbed channel	3.5 mm	75 mm
bio-inspired channel	3.5 mm	75 mm

## Data Availability

The original contributions presented in this study are included in the article material. Further inquiries can be directed to the corresponding author.
